# Atypical Tactile Perception in Early Childhood Autism

**DOI:** 10.1007/s10803-022-05570-7

**Published:** 2022-04-28

**Authors:** Svenja Espenhahn, Kate J. Godfrey, Sakshi Kaur, Carly McMorris, Kara Murias, Mark Tommerdahl, Signe Bray, Ashley D. Harris

**Affiliations:** 1 Department of Radiology, Cumming School of Medicine, University of Calgary Alberta Children’s Hospital, Office B4-512 28 Oki Drive NW, T3B 6A8 Calgary, AB Canada; 2 Alberta Children’s Hospital Research Institute, University of Calgary, Calgary, AB Canada; 3 Owerko Centre, University of Calgary, Calgary, AB Canada; 4 Hotchkiss Brain Institute, University of Calgary, Calgary, AB Canada; 5 Department of Neuroscience, University of Calgary, Calgary, AB Canada; 6 Werklund School of Education, University of Calgary, Calgary, Alberta Canada; 7 Department of Pediatrics, Cumming School of Medicine, University of Calgary, Calgary, Alberta Canada; 8 Department of Biomedical Engineering, University of North Carolina at Chapel Hill, Chapel Hill, NC USA

**Keywords:** Tactile, Sensitivity, Preschool, Touch, Tactile psychophysics, Parental questionnaires

## Abstract

We assessed different aspects of tactile perception in young children (3–6 years) with autism. Autistic and neurotypical children completed vibrotactile tasks assessing reaction time, amplitude discrimination (sequential and simultaneous) and temporal discrimination (temporal order judgment and duration discrimination). Autistic children had elevated and more variable reaction times, suggesting slower perceptual-motor processing speed and/or greater distractibility. Children with autism also showed higher amplitude discrimination and temporal order judgement thresholds compared to neurotypical children. Tactile perceptual metrics did not associate with social or tactile sensitivities measured by parent-reports. Altered tactile behavioral responses appear in early childhood, can be quantified but appear dissociated from sensitivity. This implies these measures are complementary, but not necessarily related, phenomena of atypical tactile perception in autism.

## Introduction

More than 90% of children on the autism spectrum (AS)^1^ show unusual behavioral responses to sensory stimuli that arise early (Leekam S.R., Nieto C., Libby S.J., Wing L., & Gould J., 2007; McCormick C., Hepburn S., Young G.S., & Rogers S.J., 2016) and persist across development Ben-Sasson et al., 2009; Leekam et al., 2007; Tomchek S.D. & Dunn W., 2007). These sensory differences are so prevalent that “hypo- and hyper-reactivity to sensory input” are now among the diagnostic features of autism in the restricted and repetitive behaviour domain (in the DSM-5, American Psychiatric Association, 2013). Irregularities in touch and tactile perception in particular adversely affect everyday life activities (e.g., wearing clothes or grooming) and may exacerbate the social and behavioral difficulties observed in autism (Foss-Feig et al., 2012; Hilton et al., 2010; Robertson C.E. & Baron-Cohen S., 2017). However, the neurophysiological mechanisms underlying tactile difficulties in autism are poorly understood.

Historically, research investigating tactile perception in autism has focused on subjective reports of the emotional and behavioral responses to touch observed by parents and teachers. While these provide important information about the prevalence and broad nature of tactile differences in autism and their impact on function, they do not provide information about the underlying neurophysiology. More recently, psychophysical approaches have been used to directly study tactile perception in autism (for details see review by (Mikkelsen M., Wodka E.L., Mostofsky S.H., & Puts N.A.J., 2018)). At a mechanistic level, differences in tactile perceptual metrics have been linked to reduced cortical gamma-aminobutyric acid (GABA) inhibitory function in autism (Puts N.A., Edden R.A., Evans C.J., McGlone F., & McGonigle D.J., 2011; Puts et al., 2017; Sapey-Triomphe L.A., Lamberton F., Sonié S., Mattout J., & Schmitz C., 2019; Tommerdahl M., Favorov O.V., & Whitsel B.L., 2010) and thereby are consistent with the excitatory-inhibitory imbalance hypothesis of autism (Rubenstein J.L. & Merzenich M.M., 2003). However, studies have yielded mixed results in adults and children, ranging from normal tactile perception (Güçlü et al., 2007; Ide et al., 2019; O’Riordan & Passetti, 2006) to less sensitive tactile detection (e.g., the minimum stimulus that can be perceived) and discrimination (e.g., the ability to separate two stimuli) (Fründt et al., 2017; McKernan E.P., Wu Y., & Russo N., 2020; Puts et al., 2017; Puts N.A.J., Wodka E.L., Tommerdahl M., Mostofsky S.H., & Edden R.A.E., 2014; Tavassoli et al., 2016; Wada et al., 2014) to more sensitive detection Blakemore et al., 2006; Cascio et al., 2008; Riquelme et al., 2016). It is possible that these differences result from both the heterogeneity in autism and the diversity of assessment methods used, including the different types and location of stimulation. In addition, adaptation to continuous tactile stimulation appears to be altered in both adults and children on the autism spectrum (Puts et al., 2014; Tannan V., JK H., Zhang Z., Baranek G., & Tommerdahl M., 2008; Tommerdahl et al., 2007; Tommerdahl M., Tannan V., Holden J.K., & Baranek G.T., 2008); however, imaging observations have yielded mixed results, with some reporting no altered adaptation in young autistic children (Espenhahn et al., 2020) and others suggesting reduced adaptation in autism and infants at elevated likelihood of autism (Green et al., 2015; Piccardi et al., 2021).

Although psychophysical approaches have been used in older children (> age 8 years) and adults on the autism spectrum to gain insights into the condition, no studies to date have investigated tactile perception in early childhood autism. Because tactile difficulties emerge early in autism (typically < age 3 years)(Leekam et al., 2007; McCormick et al., 2016), a better understanding of potential differences in tactile perception in autistic children during early childhood is important for informing efforts to promote better quality-of-life outcomes for autistic children (Charman T., 2019; Landa R.J., 2018; Rogers et al., 2019; Sandbank et al., 2020). However, there are practical and methodological challenges associated with structured assessments of young children which we aimed to overcome with the present study.

To this end, we have developed a customized testing battery appropriate for use in young children (Kaur et al., 2021) which uses previously validated vibrotactile tasks Mikkelsen et al., 2020; Puts et al., 2011; Puts N.A., Edden R.A., Wodka E.L., Mostofsky S.H., & Tommerdahl M., 2013; Puts et al., 2017). In this study, we have used this customized battery to assess different aspects of tactile perception in young autistic children aged 3–6 years as compared to young neurotypical children. Based on the assumption that cortical inhibition is altered in autism (Puts et al., 2017; Rubenstein J.L. & Merzenich M.M., 2003; Sapey-Triomphe et al., 2019; Tannan et al., 2008; Tommerdahl et al., 2007), we expected lower tactile discrimination task performance indicated by higher thresholds in young autistic children compared to neurotypical children, consistent with observations in older children. Given the importance of touch in early development (Cascio, 2010; Thye M.D., Bednarz H.M., Herringshaw A.J., Sartin E.B., & Kana R.K., 2018), we further hypothesized that lower performance on the tactile perceptual metrics (i.e., higher discrimination thresholds) would associate with increased parent-reported social and behavioral autistic features.

## Methods

### Participants

Two cohorts of young children aged 3–6 years were tested on a tactile battery assessing different aspects of tactile perception: 33 children on the autism spectrum (AS) and 45 neurotypical (NT) children. Participants were recruited from the Owerko Neurodevelopmental Disorder Recruitment database, the Healthy Infants and Children Clinical Research Program (HICCUP) and local community. Written informed consent in accordance with the Declaration of Helsinki was obtained from a parent/guardian of each child who themselves provided informal assent to testing. The study was approved by University of Calgary Conjoint Health Research Ethics Board (REB16-0576).

All autistic children had a prior clinician diagnosis, which often included the administration of the Autism Diagnostic Observation Schedule (ADOS) (Lord et al., 2000). Clinician diagnosis was supported by parent reports on the Social Responsiveness Scale, Second Edition (SRS-2), a quantitative measure of clinical autistic traits (Constantino & Gruber, 2005). When an autistic child scored below the cut-off on the SRS-2 (≤59T), an ADOS was administered by a research-reliable rater to confirm diagnosis. Exclusion criteria included known genetic etiology of autism (e.g., Fragile X syndrome, tuberous sclerosis), seizures at the time of study entry, a history of major head trauma or loss of consciousness of > 5 min and/or neurologic disease. Four children with autism had also been diagnosed with co-occurring attention-deficit/hyperactivity disorder (ADHD), one global developmental delay (GDD) and one was born prematurely at 27 weeks gestational age. Two children were receiving medication used to treat ADHD (e.g., Strattera, Intuniv, Vyvanse). These medications were withheld for at least 24 h prior to the study visit (when possible, and with parental consent). Neurotypical participants were excluded if they had a history of neurological, psychiatric or neurodevelopmental disorder, a history of major head trauma or loss of consciousness of > 5 min, were born prematurely (< 37 weeks), were using psychotropic medications, or scored above the cut-off on the SRS-2.

Children’s sensory processing patterns in everyday life were assessed using the Child Sensory Profile 2 (CSP-2), a standardized parent-report questionnaire (Dunn, 1999). The CSP-2 is designed to assess sensory processing based on activities of daily living (e.g., at home, at school and in the community). It classifies the sensory profile into four quadrants: seeking, registration, sensitivity and avoiding to characterise different features of sensory behaviour. For each item, parents were asked to rate their child’s response to a sensory experience on a 5-point Likert scale ranging from ‘Almost Never’ (1) to ‘Almost Always’ (5). To ensure no missing data, forms were checked for completeness by the research team during the session. Scores for all questions related to the tactile domain were summed to yield a behavioral tactile sensitivity measure (CSP-2 Touch Processing Subscale, questions 16–26). General cognitive ability of all children was measured using the brief version of the Wechsler Non-Verbal (WNV) Scale of Ability (Naglieri J.A. & Brunnert K., 2009), which allows for the assessment of individuals with limited language skills. Handedness was evaluated using a parent questionnaire adapted from Kastner-Koller and colleagues (Kastner-Koller et al., 2007).

### Tactile testing

**Fig. 1:**
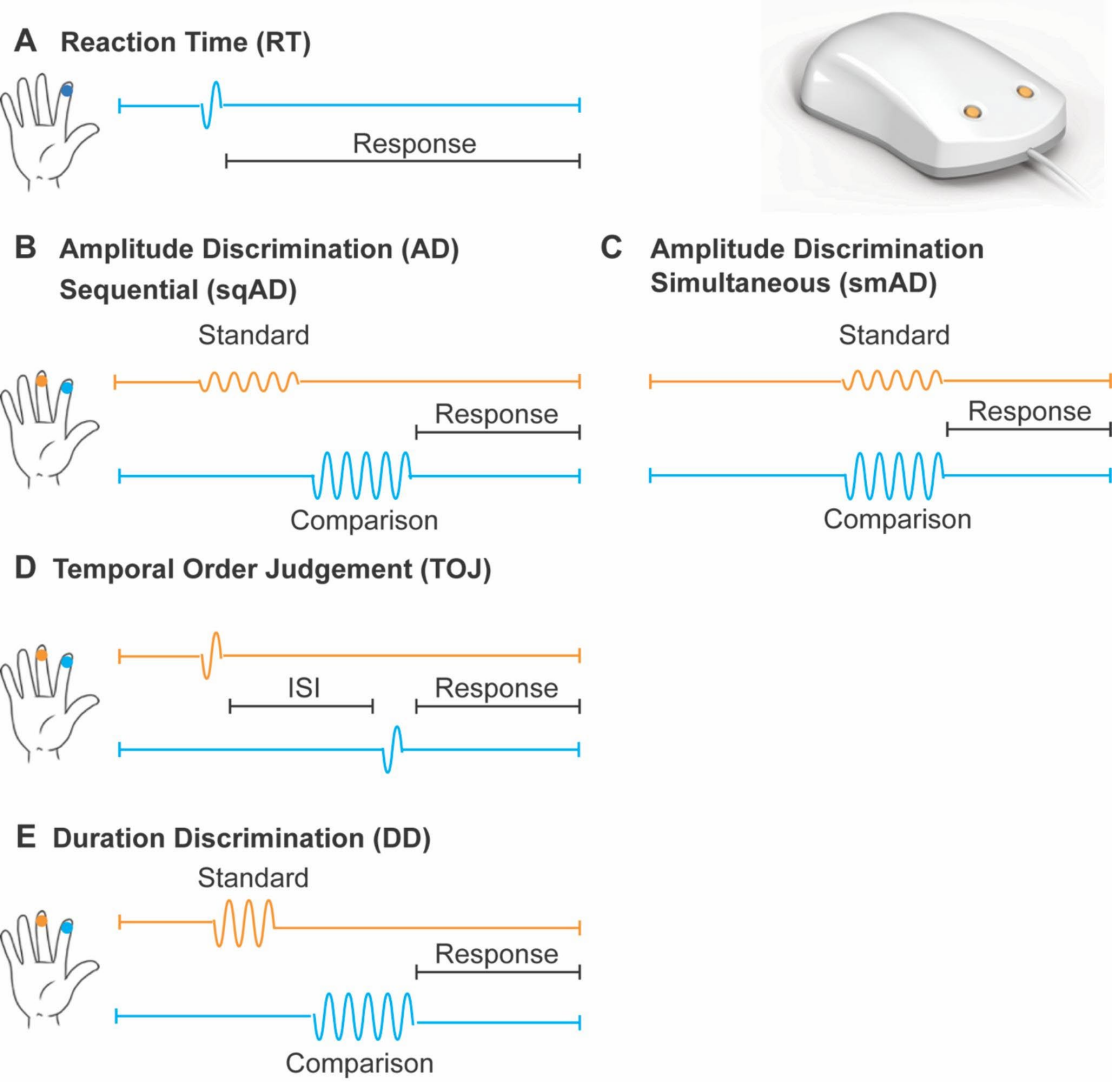
**Schematic of tactile testing battery.** A two-digit tactile stimulator was used for stimulus delivery to the left middle and index finger (top right). **A**, Reaction time (RT); **B**, Sequential and **C**, simultaneous amplitude discrimination (sqAD, smAD); **D**, Temporal order judgement (TOJ); **E**, Duration discrimination (DD). The standard stimulus is shown in orange and the comparison stimulus in blue. (Figure modified from (Kaur et al., 2021))

The tactile testing battery consisted of five different vibrotactile tasks, as shown in schematic form in Fig. 1. A two-digit tactile stimulator (Cortical Metrics, NC, USA) (Holden et al., 2012; Puts et al., 2013) was used to deliver stimuli to the glabrous skin of the participant’s left index and middle finger via cylindrical probes (5 mm diameter). All stimuli were in the flutter range (25–50 Hz) and their delivery was pseudo-randomized across the fingers. Visual feedback and data collection was performed using a Google Chromebook running CM4 software (Holden et al., 2012).

Prior to each task, at least three practice trials were administered to familiarize participants with the goal of each specific task. Participants were required to correctly respond to all three practice trials in order to proceed to the task trials. Further, throughout each task, positive reinforcement (e.g., verbal and food-related) was used to keep children motivated and engaged in the task. In order to enhance understanding of task goals, in addition to verbal instructions, visual aids were used. Feedback was provided during training but not during task trials. Participants responded via clicking the spacebar with their right hand (reaction time task) or by pointing to the respective finger (discrimination tasks). For all tasks, except reaction time, a staircase procedure was used to modulate the test parameter. Specifically, a 1 up/1 down procedure was used for the first 10 trials (difficulty was increased for correct answers and decreased for incorrect answers) and a 2 up/1 down for the remainder of the task (difficulty was increased for two correct answers and decreased for incorrect answers). The tactile testing battery took approximately 20 min to complete.

#### Reaction time (RT) Task

Suprathreshold stimuli (frequency = 25 Hz; amplitude = 300 μm; duration = 40 ms) were delivered pseudo-randomly to the left middle or index finger, and participants were asked to respond as soon as they felt a stimulus (Fig. 1A). In total, 10 trials with inter-trial interval (ITI) of 4000–7000 ms were delivered. For each participant, a measure of reaction time was calculated by averaging over the median 6 trials (e.g., excluding two fastest and two slowest trials). Reaction time variability was also calculated as the standard deviation over these median 6 trials.

#### Sequential and simultaneous amplitude discrimination (sqAD, smAD) tasks

In the sqAD task, stimuli (frequency = 25 Hz; duration = 500 ms) were delivered sequentially to the middle and index finger (inter-stimulus interval (ISI) = 500 ms) (Fig. 1B). In the smAD task, stimuli were delivered simultaneously to both fingers (Fig. 1C). In both tasks (20 trials total; ITI = 5000 ms), one finger received a standard stimulus (amplitude = 200 μm) while the other received a comparison stimulus (initial amplitude = 400 μm), and participants were asked which finger received the higher amplitude stimulus. The amplitude of the comparison stimulus decreased or increased by 20 μm for correct or incorrect responses, respectively. Amplitude discrimination thresholds (sqAD and smAD) were calculated as the mean difference in amplitude between the standard and comparison stimulus of the final 5 trials. Participants with a smaller discrimination threshold on the amplitude discrimination tasks were able to successfully discriminate between stimuli which were closer in amplitude. In addition, the amplitude difference between the sqAD and smAD thresholds was calculated for each participant (ADdiff), with positive values denoting higher sqAD thresholds than smAD thresholds, while negative values reflect higher smAD thresholds relative to sqAD thresholds. Generally, the threshold for smAD is higher than sqAD due to the respective cortical fields of each finger competing during the simultaneous processing of tactile information (Tommerdahl M., Lensch R., Francisco E., Holden J., & Favorov O.V., 2019).

#### Temporal order judgement (TOJ) Task

In this task, two stimuli (both frequency = 25 Hz, amplitude = 300 μm, duration = 40 ms) were delivered to the left middle and index finger separated temporally by a starting ISI of 150 ms (20 trials total; ITI = 5000 ms). Participants were asked to respond which finger received the first stimulus (Fig. 1D). The ISI was decreased or increased by 15% for correct or incorrect responses, respectively. TOJ thresholds were calculated as the mean of the ISI of the final 5 trials. Participants with a smaller discrimination threshold on the temporal order judgement task were able to successfully discriminate between stimuli presented closer in time.

#### Duration discrimination (DD) Task

In the DD task, two stimuli (both frequency = 40 Hz; amplitude = 300 μm) were delivered sequentially to the middle and index finger (ISI = 500 ms). One finger received a longer duration stimulus (initial comparison stimulus duration = 750 ms; standard stimulus duration = 500 ms; 20 trials total; ITI = 5000 ms) and participants were asked to respond which finger received the longer duration stimulus (Table 1E). The duration of the comparison stimulus was decreased or increased by 25 ms for correct or incorrect responses, respectively. DD thresholds were calculated as the mean difference in duration of the standard and comparison stimulus for the final 5 trials. Participants with a smaller discrimination threshold on the duration discrimination task were able to successfully discriminate between stimuli closer in duration.

### Statistical analysis

Statistical analyses were performed using SPSS (IBM SPSS Statistics, Armonk, NY, USA), custom-written Matlab (version R2017b) and RStudio (version 1.1.383) routines. All data were visually inspected before analyses. Participant’s data for individual tasks were excluded when it was reported by the experimenter that the task had not been executed properly (e.g., participant did not understand task instructions or showed poor behavioral compliance) and/or their data was more than 3 standard deviations (SD) away from the group mean (outlier). Children were also excluded if they only responded one finger for multiple stimuli (e.g., switched finger choice less than 3 times), which also indicated that they did not understand or lost focus/motivation.

Differences in tactile perception between neurotypical and autistic children were assessed using analysis of covariance (ANCOVA) for each task, with group as between-participant factor and age as a covariate given its influence on the tactile tasks (Kaur et al., 2021). Whenever group differences were found, additional analyses controlling for non-verbal IQ were conducted. A Greenhouse-Geisser correction was applied whenever Mauchly’s test indicated a lack of sphericity. Prior to ANCOVAs, Shapiro-Wilk test was used to affirm normal distribution of the data. Results were considered significant if *p* values were below 0.05. Effect sizes (η^2^ ranging between 0 and 1) and their 95% confidence intervals (CI) are given. However, this frequentist approach can only identify significant differences between groups. To test for the absence of a difference between groups, we employed Bayesian analysis in JASP (Team J., 2019), using a zero-centered Cauchy distribution with a default scale of 0.707. Bayes factor (BF_01_) was considered “anecdotal” (0–3), “strong” (10–30), “very strong” (30–100) or “extreme” (> 100) evidence for the null hypothesis (Jeffreys, 1961; Lee M. & Wagenmakers E.-J., 2014). To examine whether performance-based tactile perceptual metrics correlated with parent-reported social features and behavioral tactile sensitivity, Pearson’s correlations were calculated for both groups separately as well as across groups when controlling for group. For the group comparisons, we conducted *post-hoc* sensitivity analyses using G*Power (Faul et al., 2007) to estimate the power to detect between-group differences. All data presented in the text and tables are represented as mean±SD unless stated otherwise.

## Results

### Participant characteristics

Participant characteristics are shown in Table 1. Of the 33 children with autism recruited, 13 were unable to perform the tactile testing battery. Of the 45 neurotypical children, 3 were unable to perform the tactile testing battery, and another 2 children were excluded as they scored above the clinical cut-off for autism on the SRS-2 (> 59T). Thus, the final sample included 20 autistic children and 40 neurotypical children aged 3–6 years, which were matched for age, sex, non-verbal IQ, and handedness (statistics and *p* values are summarized in Table 1). All CSP-2 questionaires were completed. However, not all children were able to perform each vibrotactile task (Table 2 provides completion rates for each task).

As expected, the autism group showed more social impairment than the neurotypical group, with social features ranging from mildly to severely impaired, based on the SRS-2 Social Communication and Interaction T-scores (10% mild (60T–65T), 40% moderate (66T–75T), and 50% severe (≥ 76T)). Further, children on the autism spectrum exhibited significantly more parent-reported tactile sensitivity (e.g., higher scores on the CSP-2 Touch Processing subscale) than the neurotypical children. The CSP-2 measure showed high levels of internal consistency across (Cronbach’s alpha = 0.89) and within our groups (AS: Cronbach’s alpha = 0.80, NT: Cronbach’s alpha = 0.77). It is important to note that there were individual differences among children with autism, with 40% of children falling within the normal range, 20% showing ‘probable sensory differences’ (> 1 & <2 SD) and 40% showing ‘definite sensory differences’ (> 2 SD). In contrast, only 10% of neurotypical children showed ‘probable sensory differences’.

**Table 1:** Characteristics of the final sample of study participants

	NT	AS	Statistics
N	40	20	
Age [years]	5.3 ± 1.1	5.8 ± 0.8	t_(50.9)_=-1.92, *p* = 0.060
Sex (M:F)	27:13	16:4	*Χ*^*2*^ = 1.03, *p* = 0.311
Handedness (R:L:A)	38:2:0	19:0:1	*Χ*^*2*^ = 3.00, *p* = 0.223
Non-verbal IQ	106.6 ± 15.1	100.9 ± 20.3	t_(58)_ = 1.24, *p* = 0.221
Social features	45.1 ± 5.8	77.7 ± 12.4	**t**_**(23.3)**_**=-11.2**, ***p < 0.001***
Tactile sensitivity	13.46 ± 3.9	25.80 ± 8.76	**t**_**(23.12)**_**=-5.98**, ***p*** **< 0.001**

*All participants had normal or corrected-to-normal vision. Group differences in sex and handedness between children on the autism spectrum (AS) and neurotypical (NT) children were assessed using chi-square test. Non-verbal IQ was assessed using the Wechsler Non-verbal Scale of Ability, social features were assessed using Social Responsiveness Scale, Second Edition (SRS-2), Social Communication and Interaction subscale, and tactile sensitivity was measured using the Child Sensory Profile 2 (CSP-2) Touch Processing subscale. Significant effects are indicated in bold.*

### Basic processing speed

**Fig. 2:**
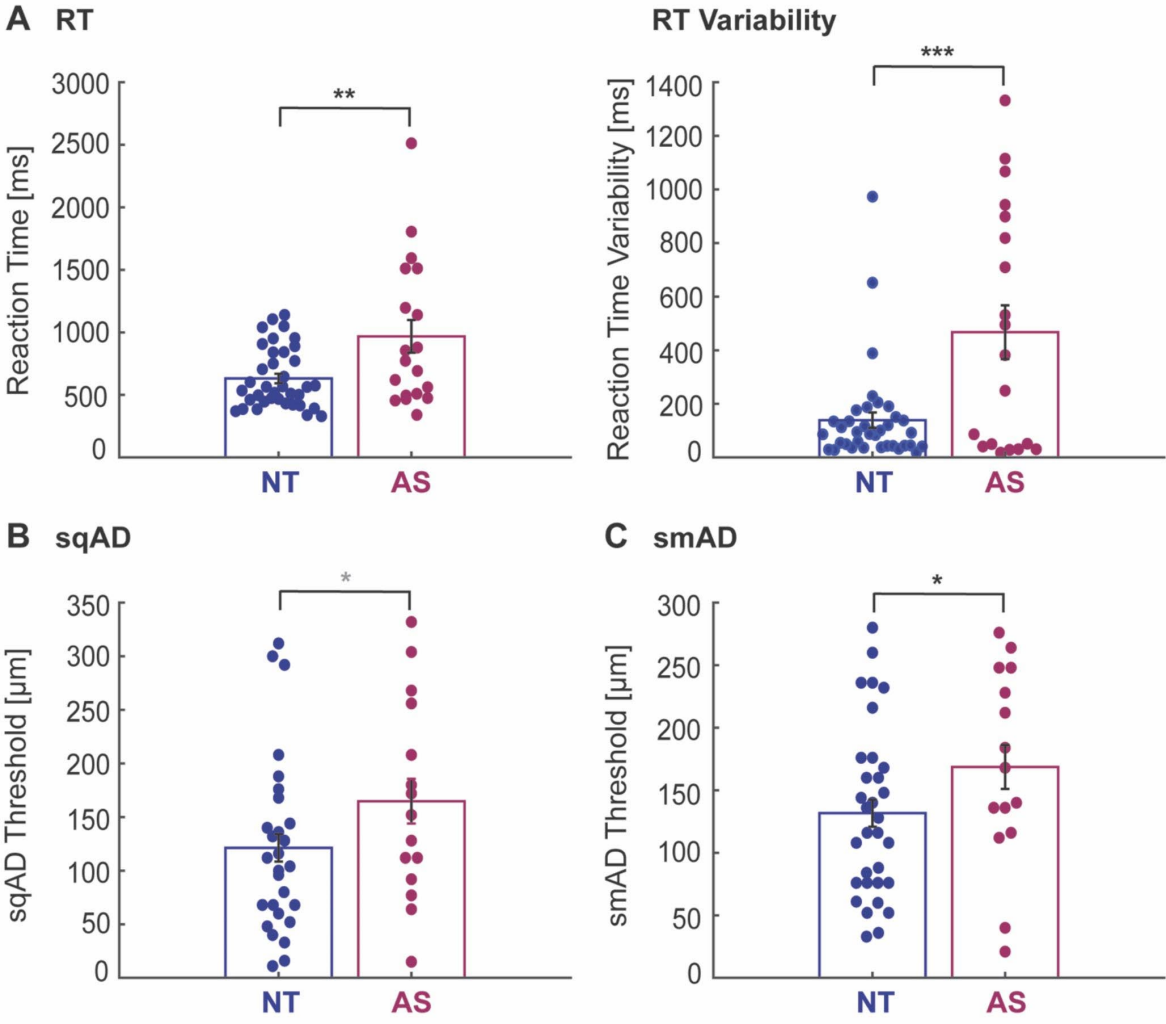
**Reaction time and amplitude discrimination measures. A**, Reaction times (RT) for children on the autism spectrum (AS, wine red) were slower and more variable compared to neurotypical (NT, blue) children. **B, C**, Sequential (sqAD) and simultaneous (smAD) amplitude discrimination thresholds were higher in children with autism than neurotypical children. **D**, The amplitude difference between the sqAD and smAD thresholds was not significantly different between groups. Dots represent individual participants and black bars represent mean ±SD across participants. All values are adjusted for age. Statistical group differences: *p < 0.05, ***p < 0.001, grey *p < 0.1 (trend), ns: non-significant

All participants were able to complete the RT task. However, reaction times for 1 autistic and 3 neurotypical children were excluded due to very slow reaction times and large variability (> 3 standard deviations as outlined in the methods section; see Table 2 for task completion rates). As can be seen in Fig. 2A and Table 3, autistic children were significantly slower to respond than neurotypical children [RT: F_(1,53)_ = 13.89, *p* < 0.001, η^2^ = 0.208, 95% CI of effect size [0.07 0.35], denoted by 36% higher RT values. In addition, children on the autism spectrum showed significantly greater variability in RT than the neurotypical group [RT: F_(1,53)_ = 19.28, *p* < 0.001, η^2^ = 0.267, 95% CI of effect size [0.11 0.41]. Including non-verbal IQ as a covariate did not change these results [RT: F_(1,51)_ = 13.36, *p* = 0.001, η^2^ = 0.208, 95% CI of effect size [0.06 0.35]; RT variability: F_(1,51)_ = 17.42, *p* < 0.001, η^2^ = 0.255, 95% CI of effect size [0.10 0.40]].

**Table 2:** Task completion rates

	NT		AS	
	**# of participants**	**% completion rate**	**# of participants**	**% completion rate**
**RT**	37	82%	19	58%
**sqAD**	28	62%	15	46%
**smAD**	32	71%	15	46%
**ADdiff**	23	51%	12	36%
**TOJ**	12	27%	9	27%
**DD**	27	60%	10	30%

*Number of participants represents neurotypical (NT) and autistic (AS) children who were able to perform each task and executed it properly. The percentage completion rate shows this as a percentage of all recruited participants in each group. RT: Reaction Time. sqAD: Sequential Amplitude Discrimination. smAD: Simultaneous Amplitude Discrimination. ADdiff: Amplitude difference between smAD and sqAD. TOJ: Temporal Order Judgement. DD: Duration Discrimination.*

### Amplitude discrimination

For the sqAD task, 32 neurotypical children and 15 autistic children were able to complete the task. However, sqAD thresholds of 4 neurotypical children were excluded due to poor execution of the task (see Table 2 for task completion rates). For the smAD task, 34 neurotypical and 18 autistic children were able to perform the task, but subsequently 2 neurotypical and 3 autistic children were excluded. There was a trend towards a significant group difference in sqAD thresholds [RT: F_(1,40)_ = 3.82, *p* = 0.058, η^2^ = 0.087, 95% CI of effect size [0.0 0.24], with the autistic children showing a 35% higher sqAD threshold than the neurotypical children (Fig. 2B and Table 3). Including non-verbal IQ as a covariate did not change this trend [F_(1,38)_ = 3.14, *p* = 0.084, η^2^ = 0.076, 95% CI of effect size [0 0.23]].

A similar pattern was observed for the smAD threshold, with the autism group showing a significantly higher smAD threshold (28% higher) compared to the neurotypical group (Fig. 2C and Table 3) [F_(1,44)_ = 4.91, *p* = 0.032, η^2^ = 0.100, 95% CI of effect size [0.01 0.25]; however, the group difference in smAD thresholds between autistic and neurotypical children did not hold when including non-verbal IQ as covariate [F_(1,43)_ = 2.21, *p* = 0.144, η^2^ = 0.049, 95% CI of effect size [0 0.18]].

While the smAD threshold is generally higher than the sqAD threshold, young children with or without autism did not show a significant difference between smAD and sqAD thersholds [NT: F_(1,21)_ = 0.53, *p* = 0.476, η^2^ = 0.024; AS: F_(1,10)_ = 0.01, *p* = 0.935, η^2^ = 0.001]. Further, the amplitude difference between sqAD and smAD thresholds was not significantly different between groups [F_(1,32)_ = 0.01, *p* = 0.922, η^2^ = 0.001, 95% CI of effect size [0 0.06]] and *post-hoc* Bayesian analysis provided anecdotal evidence for the null hypothesis of no between-group difference in the amplitude difference measure [BF_01_ = 2.96, error %<0.001].

**Table 3:** ANCOVA results for differences in tactile measures between NT and AS groups

	NT	AS	Between-group difference
**RT [ms]**	631.1±239.2	968.0±584.7	** F**_**(1,53)**_ **= 13.89**, ***p*** **< 0.001, η**^**2**^ **= 0.208 [0.07 0.35]**
**RT Variability [ms]**	139.2±183.1	467.3±449.1	** F**_**(1,53)**_ **= 19.28**, ***p*** **< 0.001, η**^**2**^ **= 0.267 [0.11 0.41]**
**sqAD [µm]**	121.3±81.5	164.8±93.0	F_(1,40)_ = 3.82, *p* = 0.058, η^2^ = 0.087 [0 0.24]*
**smAD [µm]**	131.7±68.3	168.6±78.4	** F**_**(1,44)**_ **= 4.91**, ***p*** **= 0.032, η**^**2**^ **= 0.100 [0.01 0.25]**
**ADdiff [µm]**	1.48±71.1	1.92±72.3	F_(1,32)_ = 0.01, *p* = 0.922, η^2^ = 0.000 [0 0.01]
**TOJ [ms]**	84.6±43.5	157.6±84.5	** F**_**(1,18)**_ **= 5.06**, ***p*** **= 0.037, η**^**2**^ **= 0.219 [0.01 0.44]**
**DD [ms]**	202.0±86.3	207.0±87.3	F_(1,34)_ = 0.98, *p* = 0.329, η^2^ = 0.028 [0 0.16]

ANCOVA results controlling for age. Significant effects are indicated in bold and * indicates trends. Effect sizes (η^2^ *ranging between 0 and 1) and their 95% confidence intervals (CI in square brackets) are given. RT: Reaction Time. sqAD: Sequential Amplitude Discrimination. smAD: Simultaneous Amplitude Discrimination. ADdiff: Amplitude difference between sqAD and smAD. TOJ: Temporal Order Judgement. DD: Duration Discrimination*

### Temporal discrimination

**Fig. 3:**
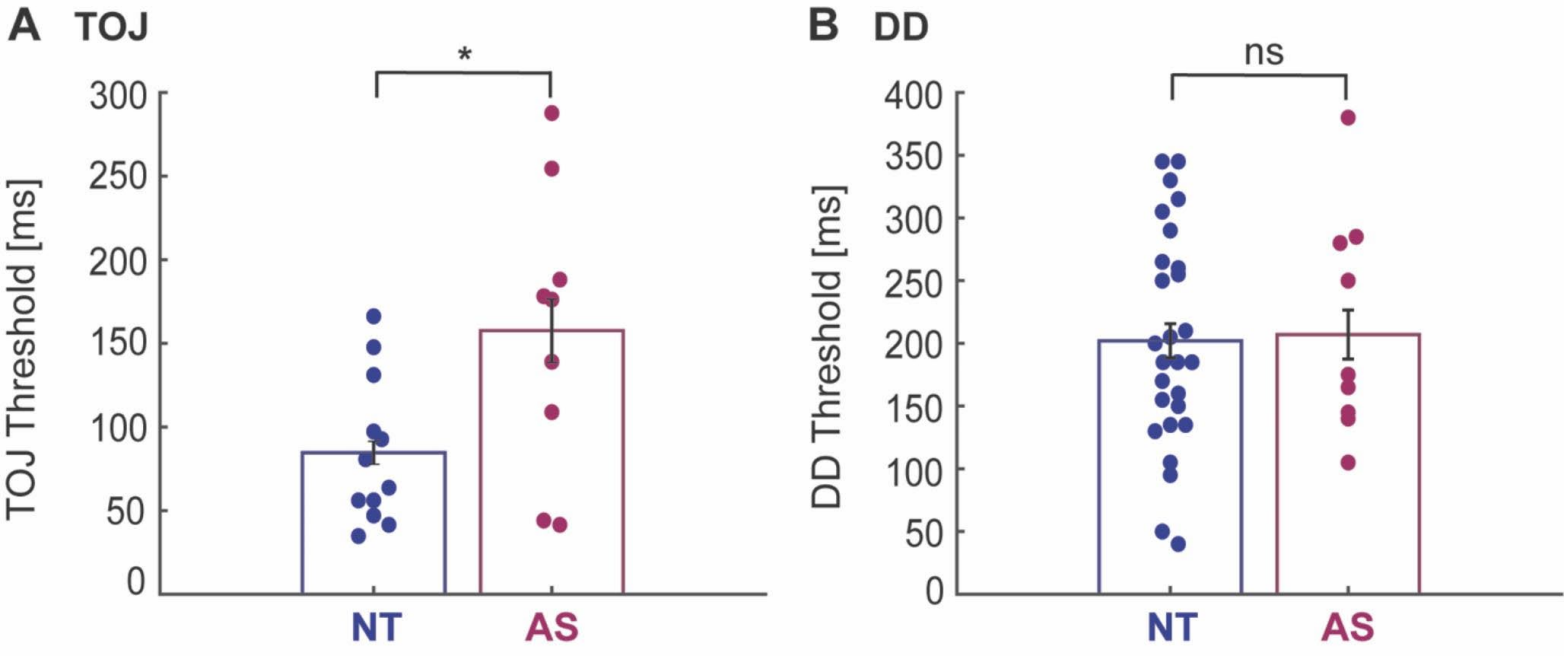
Temporal discrimination measures. A, Temporal order judgement (TOJ) thresholds were higher in children on the autism spectrum (AS, wine red) than neurotypical (NT, blue) children. B, Duration discrimination (DD) thersholds were similar between children with and without autism. Dots represent individual participants and black bars represent mean ± SD across participants. All values are adjusted for age. Statistical group differences: *p < 0.05, ns: non-significant

Due to fewer autistic children being able to perform the TOJ and DD tasks (see Table 2), statistical power to detect differences in temporal discrimination was lower and thus, the following results should be considered exploratory. The TOJ task had the lowest completion rate amongst both groups (Table 2). Seventeen neurotypical and 11 autistic children were able to perform the task, but 5 neurotypical and 2 autistic children were excluded due to poor execution of the task. TOJ thresholds, as can be seen in Fig. 3A and Table 3, were significantly different between groups [F_(1,18)_ = 5.06, *p* = 0.037, η^2^ = 0.219, 95% CI of effect size [0.01 0.44]], with children on the autism spectrum showing 86% higher TOJ threshold compared to neurotypical children. When adding non-verbal IQ as covariate, this group difference in TOJ thresholds did not hold [F_(1,17)_ = 2.39, *p* = 0.141, η^2^ = 0.123, 95% CI of effect size [0 0.35]].

For the DD task, 32 neurotypical and 15 autistic children were able to complete the task. However, 5 neurotypical and 5 autistic children were excluded. Interestingly, there was no significant difference between the neurotypical and autism group for the DD threshold [F_(1,34)_ = 0.98, *p* = 0.329, η^2^ = 0.028, 95% CI of effect size [0 0.16]] (Fig. 3B and Table 3). *Post-hoc* Bayesian analysis of this effect gave extreme evidence for the null hypothesis, suggesting that DD thresholds are indeed unaltered in young autistic children [BF_01_ = 186.5, error %=0.006].

### Confirmatory and power analysis

As a confimatory analysis, we re-analyzed differences in tactile perception between neurotypical and autistic children, using a reduced neurotypical sample (N = 20; age: 5.9±0.9 years, sex: 15 M/5F, non-verbal IQ: 109.3±12.6) to best match the two groups in terms of age, sex, non-verbal IQ and handedness [no group differences in age: t_(38)_ = 0.30, *p* = 0.764, sex: *Χ*^*2*^ = 0.14, *p* = 0.705, non-verbal IQ: t_(38)_ = 1.58, *p* = 0.124, or handedness: *Χ*^*2*^ = 3.03, *p* = 0.220].

With this reduced sample, the ANCOVA results were consistent with the above group comparisons, with slower and more variable RT, higher amplitude and TOJ thresholds, but similar DD thresholds in young children with autism compared to neurotypical children (See Supplementary Data for group averages and statistical comparisions). Similarly, when adding non-verbal IQ as covariate, only the group differences in RT and RT var were still significant, while the differences in sqAD, smAD and TOJ were no longer significant.

Lastly, *post-hoc* power analyses for between-group comparisons were conducted and are summarized in Table 4. For RT and RT var, observed power levels were greater than 0.8, while observed power for sqAA, smAD and TOJ were around 0.5. For DD, observed power was low as expected given the small sample of autistic children that were able to perform this task and results are very similar to NT.

**Table 4:** Post-hoc sensitivity analysis

	Observed power	Observed power (including IQ as a covariate)
**RT**	0.955	0.948
**RTvar**	0.991	0.984
**sqAD**	0.479	0.408
**smAD**	0.582	0.306
**AmpDiff**	0.051	0.056
**TOJ**	0.567	0.308
**DD**	0.161	0.088

### No association of tactile measures with social and sensory features

Last, we examined associations of performance-based tactile perceptual metrics with social features and behavioral tactile sensitivity as measured by the SRS-2 Social Communication and Interaction T-score and the CSP-2 Touch Processing measure. None of the tactile perceptual metrics correlated with social features or behavioral tactile sensitivity in either group, with and without accounting for non-verbal IQ (all r < 0.4 and p > 0.1). Additionally, we ran pooled (across groups) correlational analyses while controlling for group and did not find any associations between tactile perceptual metrics and either social features nor behavioral tactile sensitivity (all r < 0.5 and p > 0.1).

## Discussion

This study aimed to quantitatively compare tactile perception in early childhood (ages 3–6 years) comparing autistic and neurotypical children. Altered tactile function can result in significant challenges in autism but given its early emergence, management and coping strategies may have widespread implications. Indeed, there is a growing interest in understanding the reciprocal relationship between tactile processing and social development in autism (Robertson C.E. & Baron-Cohen S., 2017; Thye et al., 2018).

Using a customized testing battery that has previously been shown to be appropriate for use in young children (Kaur et al., 2021), our findings show altered tactile perception in early childhood autism on a number of vibrotactile tasks, which suggests reduced cortical inhibition in autism. Specifically, young children with autism showed poorer amplitude discrimination as well as poorer temporal order judgment (e.g., higher thresholds), but not duration discrimination, compared to neurotypical children. In addition, young children on the autism spectrum showed higher and more variable reaction times, suggestive of aberrant perceptual-motor processes. Finally, none of the tactile perceptual metrics associated with social features or behavioral tactile sensitivity as measured by parent-reports.

A small number of studies have reported higher amplitude discrimination thresholds in older autistic children (> 6 years of age) compared to their neurotypical peers (McKernan et al., 2020; Puts et al., 2014, 2017). Our study extends this literature by demonstrating that higher amplitude discrimination thresholds (both sequential and simultaneous) are already present at a younger age in autism.

The ability to discriminate stimuli relies on the ability to separate spatially distinct signals, which has been linked to GABA-mediated lateral inhibition (Puts et al., 2011, 2017; Sapey-Triomphe et al., 2019; Tommerdahl et al., 2010). Thus, elevated thresholds observed in our study could be explained, at least in part, by reduced GABAergic inhibition in early childhood autism, likely due to altered local circuitry (Casanova et al., 2003; Casanova et al., 2002). This general interpretation is also in line with magnetic resonance spectroscopy studies reporting reduced sensory and motor GABA levels in autism in both children and adults (Gaetz W. et al., 2014; Port et al., 2017; Puts et al., 2017; Rojas D.C., Singel D., Steinmetz S., Hepburn S., & Brown M.S., 2014; Sapey-Triomphe et al., 2019). However, despite evidence for a link of tactile perceptual metrics and age with GABA, we did not directly measure GABA, and thus, the inferences about GABAergic inhibition in this study are merely speculative.

Generally, the ability to discriminate simultaneously delivered stimuli is degraded compared to that of sequential stimuli due to competitive interactions of cortical areas representing the two fingers during simultaneous stimulation (Mountcastle, 1997; Tommerdahl et al., 2010, 2019). However, we did not observe a significant worsening of the amplitude discrimination ability in the simultaneous condition for either cohorts and the difference between sequential and simultaneous amplitude discrimination did not reveal an effect of diagnosis. While it appears that autistic children have inhibitory levels that are categorically different from neurotypical children, as discussed, it could be speculated that, irrespective of diagnosis, an immature inhibitory system in early childhood (Gaetz W. et al., 2014; Port et al., 2017; Saleh et al., 2020) results in worse discrimination ability, as shown in previous behavioural findings (Kaur et al., 2021), and that modulation of the relative timing of stimuli does not further change the threshold. Alternatively, this young age group is expected to show greater measurement noise, which may prevent detection of these subtle differences.

Altered inhibitory function in autism might also be a factor in timing perception (e.g., perceiving temporal relationships between sensory events). Consistent with some prior studies (Tommerdahl et al., 2008; Wada et al., 2014), but in contrast to others (Ide et al., 2019; Puts et al., 2014), we found that young children with autism exhibited higher temporal order judgment thresholds (e.g., less sensitive), which likely reflects reduced cortical inhibition. Rather unexpectedly, duration discrimination appeared unaffected in autism. There are many possible explanations for this differential result. These tasks rely on different cognitive processes, which likely develop at different rates and/or may be impacted differently by altered processing in autism.

Given the duration discrimination task was the last task in the testing battery, results may be also biased by participant fatigue. However, it appears that temporal order judgement is the most difficult task for children to complete based on the low completion rate across cohorts. While one could argue that this is attributable to fatigue or boredom, a higher completion rate of the last task in the testing battery (duration discrimination task) makes this explanation unlikely. Rather it could be due to the later maturation of the prefrontal cortex (Gogtay et al., 2004; Teffer K. & Semendeferi K., 2012) which is involved in working memory (Perlman et al., 2016) and temporal ordering (Takahashi et al., 2013) or difficulties in understanding the temporal terms used to explain the task (e.g., ‘Which stimulus came first’?) (Busby Grant & Suddendorf, 2011).

While group differences were detected for most metrics, caution is warranted in the interpretation of these results, as there is low statistical power given the small number of participants. This is particularly relevant for the timing perception tasks (although, a larger fraction completed the duration discrimination task compared to the temporal order judgement task). In cases of not detecting group differences, both frequentist and Bayesian analyses were performed and were consistent, which supports the finding of no group differences, however, both are subject to small sample size limitations.

Early childhood is a period of profound cortical and cognitive development and hence, variations in working memory and attention are also likely to impact perceptual abilities. Specifically, the ability to discriminate between two sequential stimuli requires comparison of both the remembered (first) and current (second) stimulus. While working memory and attentional capacity increase throughout childhood (Gathercole et al., 2004; Swanson 2017; Tamnes et al., 2013) and thus, are likely to account for some age-related improvements in tactile perception (Kaur et al., 2021), they are also likely to be impaired in autism (Ames C. & Fletcher-Watson S., 2010; Casey et al., 1993; Kercood S., Grskovic J.A., Banda D., & Begeske J., 2014; Wang Y. et al., 2017). Accordingly, our findings of atypical tactile perception may also be attributed to these differences in working memory and attention in autism.

Slower and more variable reaction times in young autistic compared to neurotypical children likely reflect slower perceptual-motor processes (e.g., information processing between brain regions relevant for tactile perception and those for motor preparation and execution) and greater inattention. Both perceptual-motor processes and inattention may improve with age due to natural progression, interventional therapy, or a combination of both (e.g., (Adamo et al., 2014; Ferraro, 2016; Puts et al., 2014). However, there is also evidence for persistent response differences in older children and adults (e.g., (Barbeau E.B., Meilleur A.A.S., Zeffiro T.A., & Mottron L., 2015; Geurts et al., 2008; Morrison et al., 2018)) illustrating the need for longitudinal studies in autism. While a group difference in reaction time existed in our early childhood sample even after we controlled for intellectual functioning, lower IQ did explain some differences in simultaneous amplitude discrimination and temporal order judgement. This might not be surprising given that intellectual functioning likely accounts for a large portion of variance in communication, stereotyped behaviors, and other prototypically autistic behaviors, which may partly contribute to the autism etiology. Conversely, reaction time relies more heavily on motor function.

Given the importance of touch in early development and for social behaviors (Cascio, 2010; Thye et al., 2018), a link between altered tactile perception and social skill levels could be expected. However, we did not find any associations between performance-based tactile perceptual and parent-reported social features or behavioral tactile sensitivity. This dissociation might not be surprising given that tactile perception assessed in a tightly controlled laboratory setting is unlikely to capture the multifaceted emotional, attentional and behavioral aspects of atypical tactile responses (as assessed by parent reports) (Cascio C.J., Woynaroski T., Baranek G.T., & Wallace M.T., 2016; Mikkelsen et al., 2018). In other words, performance-based and parent-report measures of tactile perception may measure complementary phenomena that could shed light on different aspects of atypical perception in autism. Future studies should thus employ multimodal approaches assessing both the cortical and affective responses to touch to establish a clearer picture of atypical tactile perception across the lifespan.

While our study is the first to assess tactile perception in early childhood autism, it is worth noting that psychophysical approaches present unique challenges in this population as they require a certain degree of verbal comprehension and attention, which are often affected by autism. Although we purposefully selected a testing battery with a relatively short duration (~ 20 min) and employed supportive strategies (e.g., visual aids), about 40% of all recruited autistic children were unable to perform the tactile testing battery. In comparison, 7% of neurotypical children could not perform the testing battery. Hence, the range of individuals on the spectrum that can be studied with this approach is somewhat restricted, limiting generalisability. Further, because our sample consisted of young children on the spectrum, we do not know about the developmental trajectory of atypical tactile perception. Our work examining tactile development suggests discrimination thresholds are rapidly changing in young children (Kaur et al., 2021) and where autism is often associated with developmental lags, longitudinal studies may assist to better compare and generalize results. Last, we acknowledge that our sample was relatively small and may have reduced statistical power that impacts the ability to precisely quantify effects. However, performing structured assessments in young children under the age of 6 years, and with autism, is highly challenging. Therefore, we recommend that future studies aiming to replicate these findings or precisely characterize these effects should recruit large enough samples to account for data loss due to task completion difficulties in early childhood. This study serves as an important first step in understanding the applicability of this approach to investigate tactile perceptual abilities in early childhood autism and will support future research in this field.

## Conclusions

In conclusion, this study assessed different aspects of tactile perception in young autistic children aged 3–6 years using a psychophysical approach. Findings showed altered tactile perception in early childhood autism. This may be related to reduced cortical inhibitory function in autism, though it is challenging to appropriately consider the moderating effect of IQ in this interpretation. In addition, tactile perceptual metrics did not associate with parent-reports of social or behavioral tactile features, implying that they do not measure the same phenomena. Overall, this study is an important first step for not only demonstrating the feasibility of quantitatively assessing tactile perception in early childhood autism, but also understanding tactile perceptual differences experienced by young children on the autism spectrum.

## Footnotes

1 *Note on terminology: To acknowledge different language preferences (Kenny et al., *2016; *Robison J.E., *2019*), the authors have chosen to use both person-first language (e.g., ‘individuals/children with autism) and identity-first language (e.g., ‘autistic children’) interchangeably to describe people on the autism spectrum and have made an effort to avoid ableist language (Bottema-Beutel K., Kapp S.K., Lester J.N., Sasson N.J., & Hand B.N., *2020*)*.
